# Effect of Angiotensin 1-7 Peptide Agonist AVE 0991 on Diabetic Endothelial Dysfunction in an Experimental Animal Model: A Possible Tool to Treat Diabetic Erectile Dysfunction

**DOI:** 10.7759/cureus.48770

**Published:** 2023-11-13

**Authors:** Deniz Coşkunsever, Murat Olukman, Emmanuele Jannini, Andrea Sansone, Giustino Varrassi

**Affiliations:** 1 Medical Pharmacology, Ege University, Izmir, TUR; 2 Systems Medicine, University of Rome "Tor Vergata", Rome, ITA; 3 Pain Medicine, Paolo Procacci Foundation, Rome, ITA

**Keywords:** male urology, erectile dysfunction, endothelial dysfunction, angiotensin agonists, diabetes

## Abstract

Background

The renin-angiotensin system and its metabolites are crucial in the pathogenesis and progression of complications of diabetes.

Aim

In this study, we aimed to evaluate the effect of angiotensin 1-7 non-peptide agonist AVE 0991 (576 ug/kg/day i.p.) on diabetic endothelial dysfunction.

Materials and methods

In this experimental animal study, we investigated the effects of angiotensin 1-7 non-peptide agonist AVE 0991 (576 ug/kg/day i.p.) treatment in male Wistar rats. Diabetes was created via injecting streptozotocin (55 mg/kg/i.p., single dose). Following the cavernous tissue submaximal phenylephrine contraction, relaxation responses were obtained by applying electrical field stimulation (0.5 ms, 40 V) for 15 seconds at 2, 4, 8, 16, 32, and 64 Hz, with two-minute intervals, respectively. To evaluate the effect of nitric oxide, the responses were compared by incubating with 100 mM N(gamma)-nitro-L-arginine methyl ester (L-NAME) for 20 minutes. Additionally, Y-27632 and sodium nitroprusside responses were evaluated in tissues contracted with submaximal doses of phenylephrine.

Results

Following a submaximal contraction of phenylephrine in the aorta rings, relaxation responses obtained with acetylcholine, sodium nitroprusside, and Y-27632 were impaired in diabetic rats; however, significant results were obtained with treatment. Although there was no significance between the groups in the electrical field stimulation responses, there was a significant dose-dependent difference in the treatment group in this parameter after L-NAME, sodium nitroprusside, and Y-27632 relaxation.

Conclusions

We determined that treatment with a non-peptide receptor antagonist of angiotensin 1-7, an enzyme detected in the aortic and cavernosum endothelium, may be a promising alternative for treating the complications of diabetes.

## Introduction

Diabetes is a metabolic, progressive, and chronic disease affecting millions of people all over the globe, classically featuring hyperglycemia consequent to changes in insulin secretion and/or insulin activity [1]. Diabetes dramatically affects patients’ quality of life (QoL), mainly for its complications than for the primary disease [2]. It is responsible for several complications, and the cardiovascular ones, whose pathogenetic mechanisms are still only partly understood, are the most important [3]. Endothelial dysfunction because of hyperglycemia is supposedly responsible for atherosclerosis, hypertension, retinopathy, nephropathy, diabetic feet, and other inflammatory/occlusive vascular diseases classified as micro- and macrovascular complications of diabetes [4].

An important complication of diabetes is erectile dysfunction (ED). ED is defined as the inability to obtain or maintain an erection sufficient for successful vaginal intercourse [5,6]. The DSM-5 has provided a more complete definition, which also requires the presence of the symptoms for at least six months, on at least 75% of sexual “occasions,” with associated distress, and without any other underlying medication or illness affecting sexual function [7]. In fact, while the occasional loss of erection is a common occurrence in healthy men, ED can be a source of significant individual and couple distress as well as a symptom of an otherwise silent condition [8]. Clinical ED is frequently anticipated in a subclinical form [9,10]. Among diabetic patients, ED occurs at an earlier age, and it is approximately three times more frequent than in the normal population [2].

Today, different medications are utilized in the treatment of diabetic and/or nondiabetic patients with ED [2]. First-line treatment is based on the administration of phosphodiesterase type 5 inhibitors (PDE5i). In case such treatments fail to restore erectile function, other treatments are available, such as intracavernous or intraurethral administration of vasoactive agents (mainly prostaglandin E-1) [11]. In cases where an underlying endocrine disorder can be found [12], it is mandatory to treat such conditions (e.g., by restoring testosterone levels acting on lifestyles [13]) or, if necessary, by administering testosterone treatment [2].

Various mechanisms account for ED in diabetes patients, including increased glycation end products, increased free oxygen radicals, impaired nitric oxide (NO) synthesis, upregulation of the RhoA/Rho kinase pathway, impaired cGMP-dependent protein kinase-1 expression, and neuropathic damage [2]. The presence of chronic, low-grade inflammation can potentially trigger additional mechanisms [14,15]. Such mechanisms could also possibly provide an explanation for the reported poor efficacy of PDE5i in diabetic patients [16,17]. Additionally, the psychological health status of diabetic patients is an often overlooked factor: however, men with diabetes usually have severely decreased QoL, including sexual QoL [15], and failure to recognize and treat any underlying psychological distress can potentially impair sexual health and function [18].

The renin-angiotensin system (RAS) has an important role in the regulation of blood pressure and is necessary for cardiovascular homeostasis, hydroelectric balance, and cellular functions [19]. Activation of the RAS generates complications of diabetes and thus promotes ED development and progression from subclinical to overt forms [9,20]. Previous studies also reported the presence of active RAS peptides in the corpus cavernosum tissue of the penis [21].

The increase in angiotensin-2 causes the secretion of nicotinamide adenine dinucleotide phosphate (NADPH) oxidase, free oxygen radicals, and, accordingly, the activation of the RhoA/Rho kinase pathway [22]. An increase in angiotensin-2 levels results in vasoconstriction, endothelial dysfunction, and insulin resistance [22]. These effects are reversed via angiotensin-2 antagonism and, for this reason, the effects of angiotensin-2 receptor antagonists have been investigated in ED [22].

Studies conducted in recent years on the effects of angiotensin 1-7 have identified its antiproliferative and antithrombotic effects [23]. Angiotensin 1-7 is directly formed from angiotensin-2 via angiotensin-converting enzyme 2 (ACE2). Angiotensin 1-7 exerts its effects on the vascular area through the mitochondrial assembly receptors (MAS), which have a G-coupled protein structure [23]. In recent years, angiotensin 1-7 and the MAS axis have been found to be active in various organs such as the brain, blood vessels, kidneys, and the heart. MAS facilitates the relaxation in the corpus cavernosum via NO-mediated calcium-activated potassium channels [24] and has important aspects in cardiovascular control [23].

Previous research elaborated that ACE inhibitors and AT1 receptor antagonists increase angiotensin 1-7 levels [21]. Based on this, it is thought that the ACE2, angiotensin 1-7, and MAS pathways may be a target in the treatment of cardiovascular diseases [21]. One of these targets is AVE 0991, a non-peptide, angiotensin 1-7, MAS receptor agonist. This orally active molecule creates angiotensin 1-7 mimetic effects in vessels, kidneys, the heart, and various organs [21]. At the same time, previous studies have revealed the cardioprotective effects of AVE 0991 [25].

ED has a multifactorial pathogenesis, with several contributing factors prominently featured in diabetic patients [15]. Low-grade inflammation, hypogonadism secondary to weight gain, depressed mood, higher incidence of urogenital infections, and lower urinary tract symptoms are common findings in men with type 2 diabetes mellitus (T2DM). Ultimately, these factors contribute to decreased expression of nitric oxide, causing endothelial dysfunction resulting in incomplete relaxation of the vascular smooth muscle of the corpora cavernosa.

Several authors have reported higher expression of angiotensin 2 in the penis than in the systemic circulation [21,26]. Owing to its aforementioned effects, a potential critical role for angiotensin-2 has been hypothesized in the progression of diabetic ED [26]. In this preclinical study, we aimed to elucidate if angiotensin 1-7 and non-peptide receptor agonist AVE 0991 may have potential therapeutic value, which can be used in the treatment of diabetic ED.

## Materials and methods

The protocol of this study was approved by Ege University (Izmir, Turkey) Animal Ethics Committee for the use of experimental animals (Approval No: 2010 - 99). Two-hundred fifty male Wistar rats obtained from the Guinea Pig Experimental Animals Laboratory (Ankara), which produces certified experimental animals were used. The rats were grouped in cages at 21±3°C room temperature for 12/12 day/night periods. The animals were given unrestricted standard rat chow and tap water. The study groups were 1) diabetes mellitus (n=20); 2) control group (n=20); 3) diluent group (10 Mm KOH) (n=20); 4) diabetes mellitus + AVE 0991 (576 mg/kg/day i.p.) (Sanofi-Aventis) group (n=20).

Establishment of Experimental DM and Treatment with AVE 0991

Diabetes was induced by injecting a single dose of streptozotocin (Applichem) (55 mg/kg, intraperitoneal, pH: 4.5) dissolved in sterile cold saline. Three days after the injection, blood glucose was measured from a drop of blood taken from the tail vein using a Contour TS glucometer (Bayer Medical Products, Mishawaka, IN). Rats with blood glucose above 250 mg/dl were considered diabetic. The animals were then randomly divided into four groups, with 20 rats in each group. AVE 0991 application was initiated eight weeks after the development of diabetes. AVE 0991 was administered i.p. at a dose of 576 mg/kg/day for two weeks.

Cavernosal Reactivity Experiments

At the end of the eight-week diabetes and treatment period, the rats were sacrificed via ketamine (100 mg/kg, i.p.) and xylazine (10 mg/kg, i.p.) anesthesia. The removed corpus cavernosum tissue was cleaned from the surrounding adipose tissue and was divided into 1x1x3 mm strips. The strips were suspended in parallel in 20 ml isolated organ baths with platinum tips at 2 g resting voltage and kept at resting voltage for one hour. The baths were filled with Krebs Henseleit solution at 37°C, pH 7.4, and continuously gassed with carbogen (a mixture of 95% oxygen + 5% carbon dioxide). The voltage changes from each strip were received by the isometric transducer and recorded continuously to the computer via an amplifier and digital converter board. Data analysis was performed using a registered software program.

At the end of the 60-90-minute adaptation and rest period, a single dose of KCl (120 mM) and a cumulatively increasing concentration of phenylephrine (1x10-9 - 3x10-5) were applied to the strips, respectively, and the contraction responses were recorded. In strips precontracted with submaximal concentrations of phenylephrine (10-5) thereafter, the relaxation response obtained with the following relaxant agents was recorded: 1) electrical field stimulation (EFS) (40V, 0.5ms pulse, two, four, eight, 16, 32, and 64 Hz at 15 s intervals) in the presence of atropine and guanethidine to evaluate non-adrenergic non-cholinergic neurotransmission; 2) rho-kinase inhibitor Y-27632 (1x10^-8^-3x10^-6^) [27]; and 3) sodium nitroprusside (SNP) (1x10^-11^-1x10^-5^) as an endothelium-mediated relaxant.

To reveal the mediation of NO release in the relaxation responses obtained, EFS responses in cavernosal strips were evaluated after 20 minutes of incubation with the nonselective NOS inhibitor N(gamma)-nitro-L-arginine methyl ester (L-NAME) (100 µM).

Isolated Organ Bath Study

The thoracic aorta was quickly removed and cleaned from the surrounding connective and adipose tissue. It was divided into 3-5 mm-wide rings and suspended horizontally in 20 ml isolated organ baths at a resting tension of 2 g. In each bath, a Krebs-Henseleit solution was used, which was exposed to a mixture of 95% oxygen and 5% carbon dioxide (carbogen) and heated to 37°C. The responses obtained from each ring were transferred to the recorder by means of a power-voltage transducer. Contractile responses were obtained with KCl (120 mM) and phenylephrine (1x10^-9^-3x10^-5^ M), respectively, in the vascular rings left at resting tension for approximately 60-90 minutes. Then, the concentration of phenylephrine that achieves 50% of the maximum contraction (EC50) and the concentration that achieves 80% (submaximal EC80) were calculated. Contractile responses were obtained with L-NAME (100 µM), an inhibitor of NO synthase, in the rings precontracted with the EC50 concentration of phenylephrine. After tissue rest, with acetylcholine (Ach, 1x10-9- 3x10-5 M) to examine endothelium-mediated relaxation responses in vessels contracted by submaximal EC80 phenylephrine precontraction and SNP (1x10^-9^-3x10^-5^ M) to examine smooth muscle relaxation responses without endothelium. Cumulative response curves were evaluated with Y-27632 (1x10^-8^-3x10^-6^), an inhibitor of the Rho kinase pathway, which is found in vascular smooth muscle and mediates contraction in vessels contracted by submaximal EC80 phenylephrine precontraction after tissue rest. After processing each agent, an approximately 45-minute waiting period has been implemented for the vessels to reach their basal resting tension.

Statistical Analysis

The results were given as the mean ± standard error. Contractile responses were expressed as mg of contraction, and relaxant responses were expressed as a percentage of phenylephrine precontraction. Analysis of variance (ANOVA) for repetitive data in the analysis of concentration-response curves in statistical evaluation. One-way ANOVA was utilized to evaluate body weight, blood glucose levels, or KCl responses, and the Bonferroni test was used for post-hoc analysis of the difference between groups. Statistical significance was set at p<0.05.

## Results

**Figure 1:**
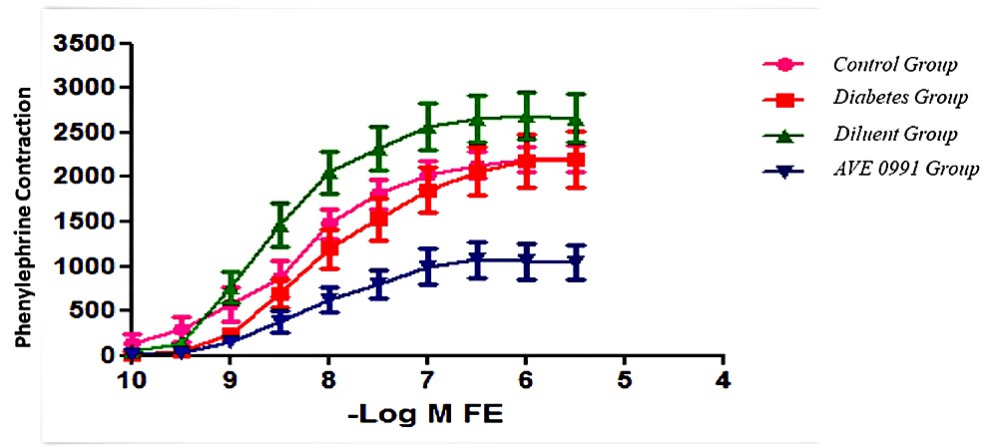
Aortic phenylephrine cumulation responses (p<0.05) The phenylephrine cumulation response decreased in diabetic rats compared to the control group and approached the control group with AVE 0991 treatment (p<0.05).

**Figure 2:**
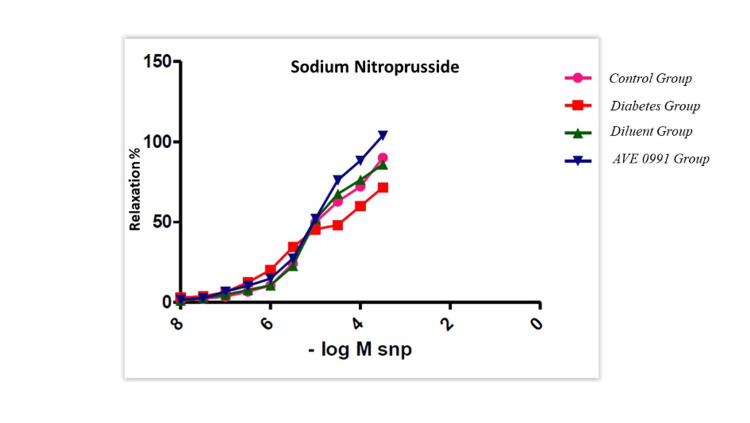
Corpus cavernosum SNP relaxation responses (p<0.05) Impaired sodium nitroprusside (SNP) relaxation responses in diabetic rats showed a significant improvement after AVE 0991 treatment compared to both diabetes and control groups, and the results were statistically significant (p<0.05).

The phenylephrine cumulation response decreased in diabetic rats compared to the control group and approached the control group with AVE 0991 treatment (p<0.05) (Figure 1). No significant response was found in the KCl responses applied to all groups. Impaired SNP relaxation responses in diabetic rats showed a significant improvement after AVE 0991 treatment compared to both diabetes and control groups, and the results were statistically significant (p<0.05) (Figure 2).

According to the EFS results obtained after Y-27632 incubation, there was a significant decrease in corpus cavernosum relaxation of diabetic rats at doses of 1x10-8-3x10-6 compared to the control group, and no curative effect of AVE 0991 treatment was detected. However, the relaxation response had decreased in the diabetes group compared to the control group, improved significantly in the AVE 0991 treatment group, and almost reached the control group levels (p<0.05).

We have applied non-adrenergic (guanitidine), non-cholinergic (atropine) relaxation to the corpus cavernosum strips. There was no significant difference between the groups in the EFS relaxation responses of 2, 4, 8, 16, 32, and 64 Hz, respectively.

**Figure 3:**
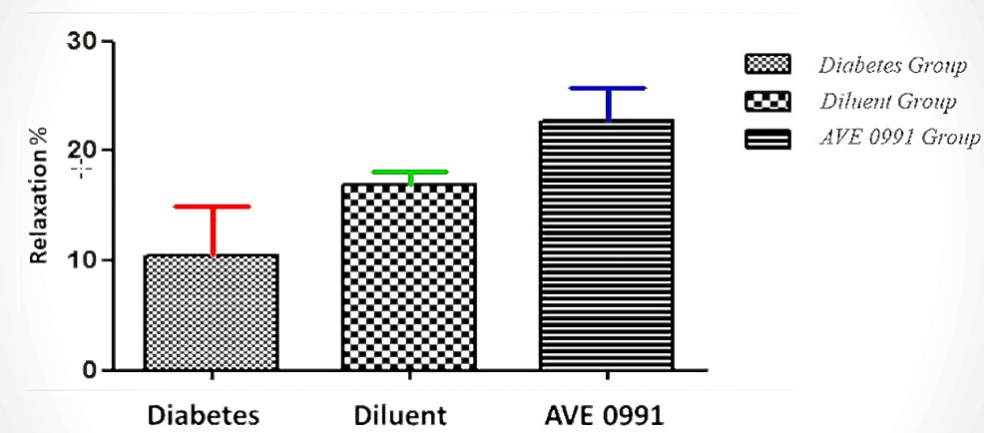
EFS relaxation responses after 20 min incubation with L-NAME in the corpus cavernosum (p<0.05) After incubation with L-NAME (NO synthesis inhibitor) together with non-adrenergic and non-cholinergic agents, a significant difference was found between the treatment group and the diabetic group in the relaxation responses (p<0.05).

After incubation with L-NAME (NO synthesis inhibitor) together with non-adrenergic and non-cholinergic agents, a significant difference was found between the treatment group and the diabetic group in the relaxation responses (p<0.05). According to the results of this research, the increased phenylephrine contractile responses in diabetes decreased statistically in the group receiving AVE 0991 (Figure 3).

**Figure 4:**
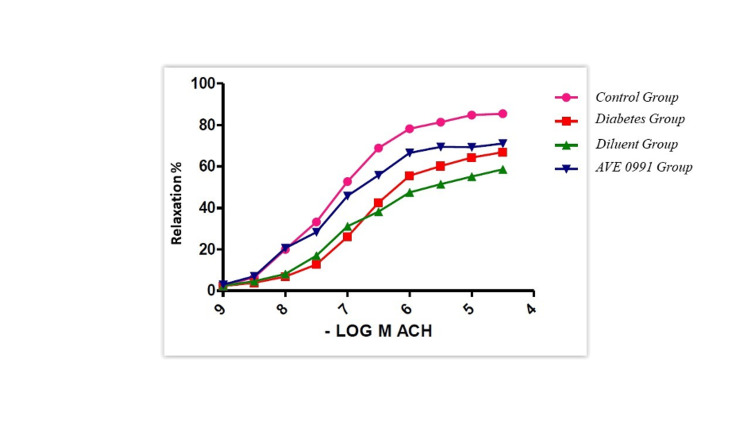
Aortic Ach relaxation responses (p<0.05) According to the results of this research, acetylcholine relaxation responses in the thoracic aorta decreased significantly in the diabetic group. AVE0991 treatment significantly ameliorated relaxation response.

Acetylcholine relaxation responses in the thoracic aorta were significantly decreased in the diabetic group (Figure 4). We found that the AVE 0991 treatment significantly improved this relaxation response. There was no significant difference between the groups in the SNP relaxation responses. Although a significant change in relaxation response was detected between the control and diabetes groups, AVE 0991 treatment did not have an ameliorating effect on this situation.

## Discussion

In this study, we have investigated the effects of angiotensin 1-7 peptide agonist AVE 0991 on erectile and endothelial dysfunction in diabetic and nondiabetic animal models and achieved significant results. Diabetes-induced endothelial/vascular dysfunction clinically accelerates the development of atherosclerosis, nephropathy, occlusive diseases, hypertension, and ED. Changes in the RAS are considered important for the development of complications of diabetes. Suppression of angiotensin II synthesis or its activity may protect against cardiovascular complications caused by diabetes or prolong its progression [25].

Previous evidence suggested that the heptapeptide angiotensin 1-7 is a potent endogenous effector hormone. The discovery of ACE2 contributed to the recognition of angiotensin 1-7 as a biologically active product of RAS. A growing number of studies showed that the biological effects of angiotensin 1-7 are controlled through the stimulation of the specific G-protein coupled receptor MAS [28,29].

In addition to angiotensin 1-7 and its MAS receptor, it has been suggested that angiotensin-converting enzyme 2 (ACE2) also forms the ACE2-Ang 1-7 MAS axis. This axis has a counterregulatory role within the ACE-angiotensin 2-AT1 axis [30]. Angiotensin 1-7 can be directly expressed from angiotensin 1 or angiotensin 2 and indirectly from angiotensin 1 in the form of an intermediate product during the secretion of angiotensin 1-9. ACE2, prolylcarboxypeptidase (PCP), and prolyl endopeptidase (PEP) can generate angiotensin 1-7 directly from angiotensin 2 [30,31].

The main enzyme and pathway involved in angiotensin 1-7 formation is ACE2-mediated angiotensin hydrolysis. However, Santos et al. [32] suggested that PEP is the main enzyme responsible for angiotensin 1-7 formation from angiotensin-2 in human coronary vessels. Angiotensin 1-7 is directly produced from neutral endopeptidase (NEP) and angiotensin 1 via hydrolysis of the Pro7-Phe8 bond with PEP [32].

RAS is the basic hormonal system responsible for the maintenance of cardiovascular homeostasis and hydroelectrolyte balance [33]. Two classes of drugs are highly effective as RAS blockers: ACE inhibitors (ACEi) act through the degradation of the RAS cascade [34], and angiotensin renin blockers-ARBs act through the blockade of AT1 receptors [35]. Blockage of the RAS with ACE inhibitors or ARBs has become one of the most successful therapeutic strategies in many cardiovascular diseases, including arterial hypertension, left ventricular systolic dysfunction, chronic heart failure, myocardial infarction, and diabetic and nondiabetic chronic kidney disease [36].

Angiotensin 1-7 increases markedly during the chronic blockade of ACE or AT1 receptors. This suggests that at least some of the effects of the blockade are mediated by angiotensin 1-7. In this way, the ACE2, Ang 1-7, and MAS axis are emerging as important possible targets for the development of new drugs for cardiovascular and renal diseases. An important step towards this goal was the discovery of the first angiotensin 1-7 analog, AVE 0991. It is an orally active non-peptide compound that mimics the effects of angiotensin 1-7 in many organs such as the vessels, kidneys, and the heart. AVE 0991 has leveraged new research areas and therapeutic possibilities in the field of cardiovascular and related diseases [36-38].

The NO/cGMP pathway is the most important intracellular mechanism in the smooth muscle relaxation necessary for erections. In the absence of the NO/cGMP pathway, the cavernosal smooth muscle cells remain in a contractile state. Apart from the adrenergic system, endothelin 1 and angiotensin 2 also contribute to the contractile response [38].

The efficacy of the RAS system has been demonstrated in the corpus cavernosum. It has been shown that angiotensin 2 was produced in physiological amounts in the corpus cavernosum. On the contrary, drugs that block angiotensin have not been found to be associated with erectile dysfunction [39].

Angiotensin 2 is not the only active peptide in the RAS system. The RAS system consists of two major branches. The first is vasoconstriction and proliferation induced by angiotensin 2 and AT1 receptors. The second is the vasodilation and antiproliferative system created by angiotensin 1-7 via MAS receptors. Based on this, it is thought that angiotensin 1-7 has an important role in penile erection. In previous literature, the presence of MAS receptors in the corpus cavernosum has been elaborated via the release of angiotensin 1-7 and NO [39,40].

AVE 0991 was first described by Wiemer et al. [41]. They stated that AVE 0991 competes for binding angiotensin 1-7 on the endothelial cell membrane with high affinity. No significant displacement of angiotensin 2 was observed for binding to AT1 or AT2 receptors. AVE 0991 is the first non-peptide synthetic compound shown to stimulate MAS. This compound mimics the effects of angiotensin 1-7 in various organs such as the blood vessels, kidneys, and the heart. AVE 0991 induced vasodilation in the aortic rings of MAS-deficient mice similar to angiotensin 1-7 [41].

Functionally, AVE 0991 stimulated the release of NO and superoxide from bovine aortic endothelial cells with efficacy similar to that observed for angiotensin 1-7. Recently, it was reported that the chronic administration of angiotensin 1-7 or AVE 0991 markedly improved the reperfusion in ischemia in the cardiac tissue of hypertensive rats. As angiotensin 1-7 was identified as a biologically active peptide with multiple cardiac effects, the results were correlated with studies showing that AVE 0991 mimics the activities of angiotensin 1-7 [41,42].

Intracavernosal injection of AVE 0991 enhances erectile response by increasing the pressure in the corpus cavernosum following electrical stimulation of the main pelvic ganglia. On the contrary, the application of A-779, a MAS receptor antagonist, completely blocks these effects. The erection facilitated by the effect of AVE 0991 is dose-dependent and completely blocked by nitric oxide synthesis inhibitor L-NAME. Angiotensin 1-7 has been shown to reverse the vasoconstrictor effects of angiotensin 2 in multiple vascular structures, including pig pial arterioles, aorta and coronary arteries, rabbit afferent arterioles, isolated pre-capillary resistance vessels, and a human mammary artery. It has been shown that both NO and prostaglandin release and increased bradykinin were associated with angiotensin 1-7-mediated vasodilation [38,39,41].

The NO-GMP pathway is accepted as the most important intracellular mechanism responsible for smooth muscle relaxation leading to an erection. Angiotensin 1-7 supported erectile response is associated with NO release. In human endothelial cells, angiotensin 1-7 has been shown to stimulate endothelial NOS phosphorylation resulting in NO production via Akt-dependent pathways. This effect is similarly created via AVE 0991, which also induces NO release. AVE 0991-induced NO release was inhibited by A-779 in MAS-transfected Chinese hamster ovary cells, but not by angiotensin type 1 or type 2 receptor antagonists. We also observed that the facilitated pro-erectile response of AVE 0991 was completely blocked by the NOS inhibitor L-NAME. These data indicate that the AVE 0991-induced erectile function is dependent on NO formation [38,42].

Phosphodiesterase 5 inhibitors are widely prescribed for the treatment of erectile dysfunction [43-45]. Cardiovascular adverse events associated with the use of phosphodiesterase 5 inhibitors have been mainly attributed to vasodilatory mechanisms. Serious cardiovascular events, including life-threatening hypotension, may also occur as a result of combined therapy of phosphodiesterase 5 inhibitors and systemic nitrates. In this study, we did not observe any fluctuation in blood pressure or heart rate in normotensive rats because of the administration of AVE 0991. Indeed, in previous studies, even higher doses of AVE 0991 have been used compared to our research with no significant changes in blood pressure or heart rate [46].

In this study, we found that Ach and SNP responses were higher in both the corpus cavernosum and thoracic aorta of rats receiving AVE 0991, compared to diabetic rats. Application of Y-27632, an inhibitor of the Rho kinase enzyme, which provides contraction by phosphorylating myosin light chain phosphatase (MLC) in smooth muscle, increased relaxation responses in rats receiving AVE 0991 treatment compared to diabetic rats both at the corpus cavernosum and the thoracic aorta. Our results confirmed previous observations in nondiabetic rats using a different experimental model [47].

Although we did not detect any significance in EFS response between the groups, we found that the application of L-NAME did not block the EFS relaxation responses in the *corpus cavernosum* and even increased relaxation response in the treatment group compared to the diabetic group. Therefore, apart from the NO-dependent relaxing effect of AVE 0991 on the corpus cavernosum, it should be considered that different mechanisms may also have an effect on relaxation.

According to the thoracic aorta isolated organ bath experiments, no significant data could be obtained between the rats treated with AVE 0991 and diabetic rats in the results of L-NAME applied after submaximal precontraction with phenylephrine, but a significant difference was found between the control group and the diabetic group [41,48].

This study has a few limitations. Other tissues with endothelium should have been studied (e.g., intestine, ureter). This would have suggested some potential explanation and therapy for gastrointestinal and urological problems typical of diabetic patients. The experiments performed limit the potential transferability of data to humans only for arteries and *corpus cavernosum*, which is extremely important for the clinical consequences, but it is limiting. Other studies should also be performed for any kind of endothelium. This study represents a good starting point.

## Conclusions

This study proves that the effects induced by AVE 0991 in the *corpus cavernosum* were comparable to angiotensin 1-7. In the used diabetic rodent models, AVE 0991 significantly improves erectile and endothelial function through MAS/NO-dependent mechanisms, which are impaired in diabetes. To the best of our knowledge, this is the first study investigating the potential use of AVE 0991 as a new target in the recovery and/or improvement of erectile and endothelial functions, consequent to diabetes. While this approach seems promising, additional data from in vivo experiments and in-human models are necessary, and further studies are therefore warranted.
